# The role of locus of control in shaping graduates’ entrepreneurial intentions: the mediating role of self-efficacy

**DOI:** 10.3389/fpsyg.2025.1664105

**Published:** 2025-09-24

**Authors:** Bereket Merkine Gebresilase, Chuanxia Zhang, Esayas Teshome Taddese, Phawani Vijayaratnam, Zebdewos Zekarias Elka, Yohannes Bisa Biramo

**Affiliations:** ^1^College of General Education, Shandong Xiehe University, Jinan, China; ^2^Faculty of Education and Liberal Arts, INTI International University, Nilai, Malaysia; ^3^Department of Psychology, Wolaita Sodo University, Sodo, Ethiopia

**Keywords:** social entrepreneurship, locus of control, self-efficacy, unemployment, Ethiopia

## Abstract

Entrepreneurship is a crucial driver of economic development in countries like Ethiopia, where high graduate unemployment and limited government job opportunities present ongoing challenges. However, little research has examined how self-efficacy mediates the relationship between internal and external locus of control and graduates’ entrepreneurial intentions. This study aimed to investigate these relationships among graduating students. Data were collected from 455 final-year students (“Mage = 20.40″, SD = 2.52), of whom 280 (61.5%) were male and 175 (38.5%) were female. The results indicated that an internal locus of control was positively associated with graduates’ entrepreneurial intentions, whereas an external locus of control was negatively related to entrepreneurial intention. In addition, self-efficacy showed a significant positive correlation with graduates’ entrepreneurial intention. Structural equation modeling further revealed that self-efficacy partially mediates the relationship between both internal and external locus of control and entrepreneurial intention. These findings underscore the importance of strengthening students’ sense of personal control and self-efficacy to enhance their motivation and capacity to pursue entrepreneurship.

## Introduction

In the dynamic landscape of the global economy, entrepreneurship is increasingly recognized as a powerful engine for economic growth, innovation, and social transformation. It plays a pivotal role in job creation, poverty alleviation, and technological advancement, and continues to gain attention from governments, educators, and researchers worldwide (Kuckertz et al., 2020; Obschonka et al., 2012; Batrancea, 2021). For developing nations like Ethiopia where poverty, youth unemployment, and limited innovation systems pose significant challenges fostering entrepreneurship among graduates is not just a policy option but a developmental necessity (Werotew, 2010; Muchie and Bekele, 2009; Batrancea and Goje, 2025). Despite its young and growing population, Ethiopia has historically recorded some of the lowest self-employment rates in Sub-Saharan Africa 12% compared to a regional average of 28% (Bosma, 2013). Recent governmental efforts, including the expansion of entrepreneurship education and the establishment of an Entrepreneurship Ministry, reflect a strong commitment to reversing this trend. Nonetheless, progress has been impeded by persistent structural issues such as political instability, low digital penetration, corruption, and high unemployment. According to recent data, urban unemployment has surpassed 1.1 million, and nearly 37% of the urban population lives below the poverty line (World Bank Group, 2022; Citaristi, 2022).

Graduates are expected to play a central role in transforming the country’s economic landscape by shifting from job seekers to job creators. However, this transition requires not only technical and managerial competencies but also the cultivation of an entrepreneurial mindset. Higher education institutions must therefore take an active role in equipping students with the attitudes, skills, and psychological readiness necessary for successful entrepreneurship (Mwasalwiba et al., 2014; Pardim et al., 2022). A central element in understanding entrepreneurial behavior is entrepreneurial intention, which refers to a person’s conscious and deliberate plan to start a business in the future (Shinnar et al., 2014; Pérez-Fernández et al., 2022). It is widely accepted as the strongest predictor of entrepreneurial action (Ajzen, 2002; Liñán and Fayolle, 2015). In developing countries, entrepreneurial intentions are especially relevant due to limited employment options and the need for economic self-reliance (Urban and Kujinga, 2017; Abaho et al., 2024). Understanding the psychological and behavioral determinants of entrepreneurial intention is thus essential for shaping effective policies and educational interventions.

Among these determinants, locus of control stands out as a critical personality trait that influences how individuals perceive opportunities and take initiative. Individuals with an internal locus of control who believe that their efforts directly affect outcomes are more likely to exhibit entrepreneurial tendencies, including risk-taking, goal orientation, and persistence (Sagita et al., 2025; Karimi et al., 2012; Şahin et al., 2019; Batrancea, 2021). This belief in personal control fosters proactive behavior and a greater willingness to navigate uncertainty qualities essential for entrepreneurship. Closely related is the concept of self-efficacy, defined as a person’s belief in their ability to perform tasks and achieve goals (Bandura and Wessels, 1997). Self-efficacy not only influences motivation and decision-making but also plays a crucial role in determining whether entrepreneurial intentions are formed and translated into action (Nowiński et al., 2019; Newman et al., 2019; Liu et al., 2019). Research shows that self-efficacy acts as a psychological bridge, enabling internal beliefs such as locus of control to impact behavioral outcomes, including entrepreneurship (Tsai et al., 2016; Sitaridis and Kitsios, 2019). It is impossible to properly comprehend psychological traits like locus of control and self-efficacy in settings like Ethiopia without taking into account the institutional and cultural milieu. Many graduates believe that career success depends on government funding rather than personal initiative because state-centered growth and public employment have historically promoted a culture of dependency (Urban and Kujinga, 2017). Similar to this, insufficient entrepreneurial infrastructure and inadequate innovation ecosystems hinder proactive people’s ability to convert their efforts into observable results, which may strengthen the external locus of control (Hartmann et al., 2022). The way that graduates view control and ability may also be influenced by collectivist orientations and familial expectations, since family networks frequently offer both financial support and risk-taking restrictions. In light of this, cultivating entrepreneurial self-efficacy may be particularly difficult because dependency norms and structural impediments can erode the conviction that one’s own hard work is enough to achieve success as an entrepreneur. In addition to extending TPB, analyzing the mediating function of self-efficacy in the Ethiopian context shows how institutional and cultural factors influence the psychological underpinnings of entrepreneurial intention.

### Locus of control and entrepreneurial intention

Locus of control is a well-established psychological construct that refers to an individual’s generalized belief about the extent to which they can control events that affect their life (Rotter, 1966). Individuals with an internal locus of control believe that their own efforts, abilities, and actions determine the outcomes they experience. In contrast, those with an external locus of control attribute outcomes to external forces such as luck, fate, or powerful others (Pinochet et al., 2023). This belief system significantly shapes motivation, behavior, and decision-making processes, particularly in the domain of entrepreneurship. Empirical studies consistently demonstrate that individuals with a strong internal locus of control are more likely to exhibit entrepreneurial tendencies, as they perceive themselves as autonomous agents capable of initiating and managing change (Alhalalmeh et al., 2025; Mueller and Thomas, 2001; Mensah et al., 2021). Internally oriented individuals are typically more proactive, persistent in the face of adversity, and more inclined to take responsibility for both successes and failures traits that are fundamental to entrepreneurial behavior (Zhao and Wibowo, 2021; Asante and Affum-Osei, 2019). These individuals also tend to engage more in opportunity recognition and risk-taking, which are key components of the entrepreneurial process.

Interestingly, while internal locus of control has been widely associated with positive entrepreneurial outcomes, some studies have found external locus of control to also correlate with entrepreneurial intention through different mechanisms. For instance, Arkorful and Hilton (2022) and Ndofirepi (2020) suggest that individuals with external locus orientations may still pursue entrepreneurship, particularly in contexts where external factors (e.g., job scarcity, economic instability) pressure individuals toward self-employment as a survival strategy. This suggests a more complex relationship in which both internal and external orientations may influence entrepreneurial intention, albeit through distinct motivational pathways (Baldegger et al., 2017; Krueger et al., 2000; Pardim et al., 2024). Within the entrepreneurship literature, entrepreneurial intention is understood as a cognitive state that precedes and predicts entrepreneurial behavior (Liñán and Fayolle, 2015). It is shaped by both intrinsic psychological traits and extrinsic environmental factors. The concept of entrepreneurial competence is a composite of personality traits, knowledge, skills, and abilities has been proposed to explain why individuals respond differently to the same environmental stimuli (Bird, 2019; Mitchelmore and Rowley, 2010). Locus of control is increasingly recognized as a foundational element of this competency framework (Lee et al., 2016; Jain, 2011).

However, while entrepreneurial abilities and competencies can evolve through learning and experience (Tittel and Terzidis, 2020; Shane and Venkataraman, 2000), locus of control tends to be relatively stable, as it is shaped by long-standing cultural, social, and value-based influences (Hartmann et al., 2022). This stability underscores its predictive power in early-stage career decision-making, such as the intention to pursue entrepreneurial ventures immediately after graduation. Despite the growing body of literature, limited research has explored the combined roles of both internal and external locus of control in shaping the entrepreneurial intentions of new graduates, particularly in developing countries where contextual pressures and opportunity structures differ significantly from those in more developed economies. Moreover, the mechanisms through which these personality traits translate into entrepreneurial intentions such as via self-efficacy remain under-investigated in such settings. Addressing this gap can provide a more nuanced understanding of how young people navigate the entrepreneurial landscape based on their internal psychological orientation.

### Self-efficacy as a mediator

Self-efficacy refers to an individual’s belief in their capacity to successfully perform tasks and achieve goals, particularly when confronted with challenges (Bandura and Wessels, 1997). Within entrepreneurship research, entrepreneurial self-efficacy (ESE) has emerged as a critical domain-specific construct, denoting confidence in one’s ability to successfully initiate and manage entrepreneurial activities (Santos and Liguori, 2020; Liñán and Fayolle, 2015; Almeida et al., 2023). A growing body of literature emphasizes the mediating role of ESE in transforming personal characteristics into entrepreneurial intentions (EI), particularly among university students and recent graduates (Buli and Yesuf, 2015; Doanh and Bernat, 2019). Locus of control (LoC), the extent to which individuals believe they have control over outcomes in their lives is one such personality trait strongly associated with self-efficacy. Individuals with a high internal locus of control perceive themselves as agents of change and believe outcomes result from their own effort and decisions, which in turn boosts their entrepreneurial confidence (Kusmintarti et al., 2014; Fatoki, 2019). In contrast, those with an external locus of control attribute outcomes to luck, fate, or powerful others, and are less likely to develop the self-efficacy needed for entrepreneurial pursuit (Sarwoko, 2020).

According to Social Cognitive Theory, internal control beliefs shape self-efficacy by reinforcing individuals’ perceptions of capability in the face of challenges, thereby enhancing the likelihood of forming strong entrepreneurial intentions (Bandura and Wessels, 1997). Empirical research supports this cognitive pathway. For instance, Zaremohzzabieh et al. (2019) found that ESE mediates the influence of psychological traits, including locus of control, on entrepreneurial intention among university students. Similarly, Al-Qadasi et al. (2023) showed that self-efficacy partially explains how personality and social influence variables translate into entrepreneurial action readiness. These findings are consistent with the Theory of Planned Behavior, which highlights perceived behavioral control a construct analogous to self-efficacy as a proximal determinant of behavioral intentions (Ajzen, 2002). The mediating role of self-efficacy is especially relevant for graduates navigating the uncertain transition from education to self-employment. During this period, individual beliefs about competence can play a pivotal role in shaping the motivation to pursue entrepreneurial ventures, particularly when internal control beliefs are already strong. Recent cross-cultural studies also confirm the robustness of this mediational mechanism in diverse educational and economic settings (Entrialgo and Iglesias, 2020; Nowiński et al., 2019).

Although perceived behavioral control is emphasized by the TPB as a significant factor in determining entrepreneurial intention, the psychological dispositions that underlie these control beliefs are not explained. The origins of control beliefs in entrepreneurial situations are one of the TPB framework’s less well-developed mechanisms, which this study elucidates by establishing locus of control as a distal antecedent of self-efficacy. A fresh addition is also made by placing this extension in the context of Ethiopian graduates. Self-efficacy and locus of control are two examples of internal psychological resources that may be particularly important in determining entrepreneurial intents in an environment where structural unemployment and a lack of entrepreneurial ecosystems limit external opportunities. Thus, the present study advances TPB both theoretically, by integrating personality dispositions into the formation of perceived control, and contextually, by adapting the framework to a low-income, high-unemployment environment.

### Current study and hypothesis

Unemployment remains a critical sociolect-economic challenge in present-day Ethiopia, with thousands of university graduates struggling to find jobs each year, as documented by various zonal and regional reports. This widespread issue affects virtually every part of the country and poses a serious threat to national development. In response, entrepreneurship is increasingly recognized as a promising solution to alleviate graduate unemployment by encouraging self-employment and innovation. However, becoming an entrepreneur requires more than just technical skills it demands psychological resilience, self-belief, and a proactive mindset. Despite this, there is limited understanding of how psychological factors, particularly locus of control and self-efficacy, influence the motivation and intention of graduates to engage in entrepreneurial activities. Moreover, few studies have examined how self-efficacy mediates the relationship between both internal and external locus of control and entrepreneurial intention within the Ethiopian context.

**Figure 1:**
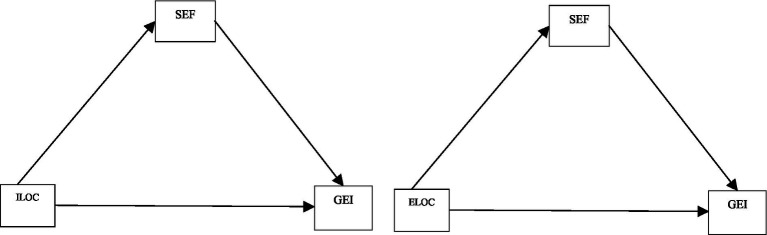
The study model ILOC refers internal locus of control, ELOC -external locus of control, SE- Self-efficacy, GEI refers graduate’s entrepreneurship intention.

This study aims to fill these gaps by exploring how locus of control serves as a key psychological factor influencing graduates’ willingness to pursue self-employment rather than relying solely on government job opportunities. Furthermore, it investigates the mediating role of self-efficacy in the relationship between internal/external locus of control and entrepreneurial intention. The conceptual framework of the study is illustrated in the following model diagrams (Figure 1).

### Hypothesis of the study

To achieve the aim of the research the following hypotheses were tested:

*H1*: Internal locus of control is positively correlated with graduates entrepreneurship intention.

*H2*: External locus of control is negatively correlated with graduates entrepreneurship intention.

*H3*: Locus of control is positively correlated with graduates self-efficacy.

*H4*: Self-efficacy mediates the association between locus of control and graduates entrepreneurship intention.

## Methods

This study utilized a cross-sectional research design to examine the mediating role of self-efficacy in the relationship between locus of control and graduate’s entrepreneurship intention. It is important to acknowledge that while mediation is traditionally tested using longitudinal designs to establish causal pathways (Maxwell and Cole, 2007); mediation analysis using cross-sectional data was conducted in this study to explore potential indirect relationships. The interpretation of mediation results is therefore exploratory and should be viewed with caution, recognizing the inherent limitations of causal inference in cross-sectional designs (Rucker et al., 2011).

### Study area

The study was carried out in Wolaita Sodo, the administrative center of Ethiopia’s Southern Region, located about 385 kilometers south of Addis Ababa. Wolaita Sodo University, a government institution, serves roughly 35,000 students enrolled in seven colleges and three schools. Its diverse student body is drawn from all regions of the country, making the university a reasonable reflection of Ethiopia’s broader population. The university operates across three campuses: two are based in Wolaita Sodo city itself, while the third is in Tercha town, which is part of the Dawuro Zone and lies 90 kilometers from Wolaita Sodo.

### Sample and sampling techniques

The sample for this study was chosen through a multi-stage process. First, purposive sampling was applied to focus specifically on graduating students, as the study aimed to explore how self-efficacy mediates the relationship between locus of control and entrepreneurial intentions among graduates. Next, the student population was stratified by college and gender to account for notable variations in enrollment across different colleges. To achieve equal representation and minimize selection bias, simple random sampling was then conducted within each stratum using the lottery method. This approach is effective for producing comparable groups that accurately reflect key demographic variables (Amin, 2005). A total of 455 (2022 graduating class students) participated in the study (M_age = 20.40, SD = 2.52), drawn from seven colleges and three schools. Of these, 280 (61.5%) were male and 175 (38.5%) were female. The inclusion criteria was graduating class student of Wolaita Sodo University in 2022 academic calendar. The final sample size was determined using the Krejcie and Morgan (1970) sampling table, which is widely recognized for its practical guidelines and has been consistently validated by researchers for determining appropriate sample sizes for finite populations.

### Procedures

Two senior language instructors, both native speakers and faculty members in language departments, translated the questionnaire into the country’s official language. In line with Brislin’s (1986) guidelines, a translation and back-translation procedure was employed to ensure conceptual equivalence of the survey instruments. Ethical approval for the study was granted by the university’s research ethics committee. After recognizing the relevance and potential contributions of the research, the host university authorized its implementation. Participants were assured of complete anonymity, informed that participation was voluntary, and reminded of their right to withdraw or decline involvement at any stage.

## Measures

### Locus of control

Both internal and external locus of control were assessed using a 10-item scale developed by Chen et al. (1998). Example items include: *“I am usually able to protect my personal interests”* (internal locus of control) and *“When I get what I want, it’s usually because I’m lucky”* (external locus of control). Participants rated each statement on a five-point Likert scale ranging from *strongly disagree* to *strongly agree*. To ensure the reliability of the scale, Cronbach’s alpha was calculated and demonstrated a high level of internal consistency (*α* = 0.84). In addition, a confirmatory factor analysis (CFA) was performed to evaluate the fit of the translated locus of control measure to the data. The CFA results indicated a good model fit, with the following fit indices: χ^2^/df = 2.47, CFI = 0.96, TLI = 0.95, SRMR = 0.04, and RMSEA = 0.073.

### Self-efficacy

Self-efficacy was evaluated using a revised inventory consisting of 10 items developed by Schwarzer and Jerusalem (1995), such as “I can always manage to solve difficult problems if I try hard enough.” Participants responded on a four-point scale, ranging from “not at all true” to “exactly true.” Items were averaged, with high scores indicating high levels of self-efficacy. To ensure the reliability of the measure, Cronbach’s alpha was calculated, demonstrating a high level of internal consistency (Cronbach’s alpha coefficient of 0.89). Additionally, confirmatory factor analysis (CFA) was conducted to assess the fit of translated self-efficacy measure to the data. The results of the CFA indicated a good fit, as evidenced by the following fit indices: χ2 /df = 3.04, CFI = 0.95, TLI = 0.95, SRMR = 0.03, RMSEA = 0.05.

### Entrepreneurship intentions

Entrepreneurial intention was assessed using the 6-item sub-scale from the Entrepreneurial Intention Questionnaire (EIQ) developed by Linán and Chen (2009). Respondents rated their agreement on a 7-point Likert scale (1 = strongly disagree to 7 = strongly agree) items like “I will make every effort to start and run my own business.” Higher scores reflected stronger entrepreneurial intention. To ensure the reliability of the measure, Cronbach’s alpha was calculated, demonstrating a high level of internal consistency (Cronbach’s alpha coefficient of 0.88). Additionally, confirmatory factor analysis (CFA) was conducted to assess the fit of translated entrepreneurship intentions measure to the data. The results of the CFA indicated a good fit, as evidenced by the following fit indices: χ2 /df = 3.12, CFI = 0.97, TLI = 0.96, SRMR = 0.02, RMSEA = 0.05.

### Data analysis

To address potential multicollinearity, all continuous variables were standardized prior to analysis. Descriptive statistics and bivariate Pearson correlation coefficients were computed for each study variable to examine initial relationships. Next, structural mediation models were specified to test whether self-efficacy mediates the relationship between locus of control and graduates’ entrepreneurial intention. The significance of indirect effects and the corresponding confidence intervals (CIs) were estimated using 5,000 bootstrap samples. All analyses were performed using Hayes’ PROCESS macro (version 4.0) in SPSS version 25.0.

## Results

### Preliminary analysis

Table 1 displays the means, standard deviations, and Pearson correlation coefficients for all key study variables. The correlation analysis revealed that internal locus of control was positively correlated with graduates’ entrepreneurial intention (*r* = 0.38, *p* < 0.01), whereas external locus of control was significantly and negatively associated with entrepreneurial intention (*r* = −0.29, *p* < 0.01). In addition, internal locus of control showed a positive correlation with graduates’ self-efficacy (*r* = 0.33, *p* < 0.01) and a negative correlation with external locus of control. Furthermore, self-efficacy was significantly and positively related to graduates’ entrepreneurial intention (*r* = 0.42, *p* < 0.01).

**Table 1:** Descriptive and bivariate correlations results of study variables.

Variable	1	2	3	4	5	6	7
Gender	-						
College/school	0.07	-					
Age	0.03	0.09	-				
ILOC	0.23*	0.21	0.13	-			
ELOC	0.08	-	0.02	−0.42**	-		
SE	0.05	0.31	0.04	0.33**	−0.25*	-	
GEIN	0.012	0.12*	0.03	0.38**	−0.29**	0.42**	-
M	-	-	-	2.67	2.82	2.53	2.47
SD	-	-	-	0.76	0.91	0.52	0.55

“Gender, male/female; college/school, student belongs to; ILOC, internal locus of control; ELOC, External locus of control; SE, Self-efficacy; GEIN, Graduates entrepreneurship intention ** *p* < 0.01.

### Construct validity and reliability

We looked at Average Variance Extracted (AVE), Composite Reliability (CR), and the Heterotrait-Monotrait ratio (HTMT) to make sure that the study measures were both reliable and different. All constructs exhibited sufficient internal consistency (CR = 0.70) and convergent validity (AVE ≥ 0.50), as seen in Table 2. All construct pairs had HTMT values less than 0.85, indicating discriminant validity. The constructs assessed in this study are psycho-metrically sound and appropriate for further mediation investigations, according to these findings.

**Table 2:** Construct validity and reliability.

Construct	AVE	CR	HTMT (vs other constructs)
Locus of Control	0.58	0.91	0.53 (vs SE) / 0.41 (vs EI)
Self-Efficacy	0.67	0.88	0.42 (vs LOC) / 0.46 (vs EI)
Entrepreneurial Intention	0.60	0.92	0.47 (vs LOC) / 0.51 (vs SE)

AVE, Average Variance Extracted; CR, Composite Reliability; HTMT, Heterotrait-Monotrait ratio.

### Mediation analysis

The mediation analysis reveals that self-efficacy plays a pivotal role in linking locus of control to entrepreneurial intention. Specifically, an internal locus of control demonstrated both direct (*β* = 0.38, *p* < 0.001) and indirect effects through self-efficacy (*β* = 0.14, 95% CI [0.221, 0.432], *p* < 0.001) on entrepreneurial intention. Although the indirect pathway was smaller than the direct effect, it accounted for approximately 27% of the total influence, indicating that self-efficacy serves as a crucial mechanism through which internal control beliefs translate into entrepreneurial motivation. Conversely, an external locus of control negatively impacted both entrepreneurial intention (*β* = −0.29, *p* < 0.001) and self-efficacy, producing a significant indirect effect (*β* = −0.105, 95% CI [−0.212, −0.183], *p* < 0.001). This mediated effect represented about 26% of the total negative influence, suggesting that external control perceptions not only directly reduce entrepreneurial aspirations but also erode self-confidence, further dampening motivation.

Beyond mediation, the findings hint at self-efficacy’s potential moderating role in contexts with structural constraints. In Ethiopia’s challenging environment characterized by youth unemployment, limited financial access, and bureaucratic hurdles graduates with high self-efficacy may maintain stronger entrepreneurial intentions despite these barriers. In contrast, those with low self-efficacy might become disproportionately discouraged. These observations suggest promising directions for future research, particularly in examining a moderated mediation framework to assess how self-efficacy’s buffering effect varies under different levels of institutional and economic pressure.

**Figure 2:**
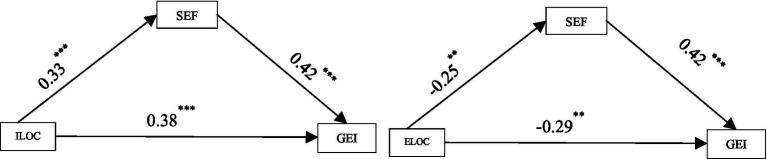
Mediation summary. Mediation was tested using Hayes’ PROCESS macro (Model 4), which evaluates indirect effects through bootstrapping rather than global model fit indices. Accordingly, model comparison was based on whether the direct effect remained significant after including the mediator, consistent with recommended practice for PROCESS analyses.

To formally evaluate whether partial mediation provided a better explanation than full mediation, we compared the size and significance of the direct effects after including the mediator. For both internal and external locus of control, the direct paths to entrepreneurial intention remained significant alongside the indirect effects via self-efficacy. This pattern supports a partial mediation model rather than a fully mediated one, indicating that locus of control influences entrepreneurial intention both directly and indirectly through self-efficacy. Notably, while internal and external locus of control are conceptual opposites, their indirect effects through self-efficacy were remarkably similar in magnitude (both around 25–27% of the total effect). This symmetry underscores the robustness of self-efficacy as a mediating psychological mechanism (Figure 2).

## Discussion

Ethiopia, like many developing countries, is grappling with a rising number of unemployed and underemployed university graduates. Despite government efforts to promote entrepreneurship through various policies, programs, and institutional support, the entrepreneurial intentions among Ethiopian youth remain relatively low (Mulugeta, 2010; Werotew, 2010). This persistent gap between policy and practice can be attributed to several cultural and structural factors. For instance, societal attitudes continue to favor stable government employment over entrepreneurial ventures, and a lack of entrepreneurial role models further discourages innovation and risk-taking. In this context, understanding the psychological and motivational determinants of entrepreneurial intention becomes critical (Batrancea and Goje, 2025). Among these, locus of control and self-efficacy have emerged as pivotal factors. This study investigated the complex relationship between locus of control and entrepreneurial intentions among recent graduates, with a particular focus on the mediating role of self-efficacy. The findings have both theoretical significance and practical implications for policymakers, educators, and development practitioners working to address youth unemployment.

### Internal locus of control and entrepreneurial intention

The results revealed that an internal locus of control is positively and significantly associated with both entrepreneurial intention and self-efficacy. This indicates that individuals who perceive outcomes as the result of their own actions are more likely to believe in their capabilities (self-efficacy) and are more inclined to pursue entrepreneurial endeavors. These individuals tend to be proactive, resilient in the face of challenges, and accountable for their successes and failures all of which are essential traits of successful entrepreneurs. This finding aligns with previous research that has consistently demonstrated a strong relationship between internal locus of control and entrepreneurial intention (Zhao and Wibowo, 2021; Asante and Affum-Osei, 2019). Theoretically, this supports Bandura and Wessels’s (1997) social cognitive theory, which emphasizes the role of personal agency and self-belief in shaping behavior and motivation.

### External locus of control and entrepreneurial intention

Conversely, the study found a negative relationship between external locus of control and entrepreneurial intention. Graduates who attribute outcomes to luck, fate, or external circumstances are less likely to develop strong self-efficacy and are therefore less inclined to take entrepreneurial risks. Such individuals may avoid situations that require personal initiative, responsibility, and persistence qualities that are critical for entrepreneurship. This finding is consistent with several previous studies (e.g., Arkorful and Hilton, 2022; Pardim et al., 2024; Almeida et al., 2023) but contrasts with others (Ndofirepi, 2020), suggesting that cultural context and measurement differences may account for these discrepancies. In Ethiopia’s context, where external factors such as economic instability and bureaucratic hurdles are perceived as major barriers, this external orientation may be more pronounced and detrimental to entrepreneurial ambition.

### Self-efficacy and entrepreneurial intention

A third major finding is the positive and significant relationship between self-efficacy and entrepreneurial intention. Graduates who have a strong belief in their ability to achieve goals and manage challenges are more likely to express intentions to start their own businesses. This underscores the central role of self-efficacy as a motivational driver for entrepreneurial behavior. From a practical standpoint, this suggests that developing students’ self-efficacy through entrepreneurship education, mentoring programs, and experiential learning opportunities can significantly boost their willingness to engage in entrepreneurial activities.

### The mediating role of self-efficacy

The final and most critical contribution of this study is the discovery that self-efficacy partially mediates the relationship between both internal and external locus of control and entrepreneurial intention. This means that while locus of control directly affects entrepreneurial intention, a significant portion of this effect operates through self-efficacy. This mediating relationship highlights the dynamic interaction between belief systems and motivation: even if individuals have an internal orientation, without confidence in their abilities, their entrepreneurial intentions may not be realized. Similarly, even externally oriented individuals can be motivated toward entrepreneurship if their self-efficacy is adequately strengthened.

The study’s findings suggest that self-efficacy serves as a mediating factor between locus of control and entrepreneurial intention. However, these results must be interpreted within Ethiopia’s unique sociocultural and institutional context. In a labor market where many graduates perceive career success as dependent on external factors such as government employment opportunities or family support prevailing attitudes often reflect an external locus of control (Urban and Kujinga, 2017). In contrast, individuals with an internal locus of control, who attribute outcomes to their own efforts and initiative, may exhibit stronger entrepreneurial intentions, as their mindset diverges from the dominant dependency norms. Furthermore, the mediating role of self-efficacy underscores the structural challenges facing entrepreneurship in Ethiopia, including weak innovation systems, limited financial access, and reliance on informal kinship networks (Hartmann et al., 2022). Even when young graduates believe in their own agency, these systemic barriers may undermine their confidence in their entrepreneurial capabilities. By highlighting how psychological factors like locus of control and self-efficacy are shaped and often constrained by broader institutional and cultural forces, this study extends the theoretical framework of the Theory of Planned Behavior.

### Practical implications

The findings suggest that fostering self-efficacy and an internal locus of control can enhance graduates’ entrepreneurial intentions. However, for these psychological factors to translate into action, interventions must be embedded within institutional and policy frameworks. First, universities should integrate experiential learning methods such as business simulations, project-based coursework, and startup competitions into their curricula. These approaches allow students to practice decision-making, take calculated risks, and receive constructive feedback, thereby reinforcing their sense of agency and self-belief. Second, mentor-ship initiatives and peer-learning networks could connect students with successful entrepreneurs, helping to counteract dependency mindsets, reduce fear of failure, and provide real-world role models. Third, establishing campus-based incubators and pilot venture programs would offer structured support for student-led startups, enabling hands-on entrepreneurial experience in a low-risk environment.

At the policy level, reforms should align educational practices with the competency-based objectives of the Ministry of Education and the entrepreneurial development goals of the Ministry of Youth. For instance, incorporating key psycho-social skills such as resilience, opportunity recognition, and proactive behavior into national graduate competency frameworks would harmonize individual psychological development with institutional expectations. Ultimately, this study underscores that boosting entrepreneurial self-efficacy among Ethiopian graduates demands not only shifts in personal mindset but also systemic innovations in education and policy.

### Limitations and future directions

While this study provides valuable insights into the relationships among locus of control, self-efficacy, and entrepreneurial intentions, several limitations should be noted. First, the cross-sectional design restricts our ability to infer causality or examine changes in psychological constructs over time. Second, reliance on self-reported data may introduce biases, including social desirability and recall bias. Third, the study sample consisted solely of university graduates, limiting the generalizability of the findings to broader populations.

To address these limitations and extend this line of research, future studies could adopt longitudinal designs to track how locus of control and self-efficacy evolve after graduation and how these changes impact entrepreneurial behavior. Experimental or intervention-based research is also recommended to evaluate the effectiveness of educational programs or training in enhancing self-efficacy and fostering an internal locus of control. Additionally, employing mixed-methods or multilevel analyses could provide richer insights into contextual influences, such as differences across faculties, academic disciplines, or geographic regions, allowing for a more nuanced understanding of the psychological and environmental factors that shape graduates’ entrepreneurial intentions.

## Data Availability

Publicly available datasets were analyzed in this study. Data are available on request to the authors.
